# Targeted Therapy in Melanoma and Mechanisms of Resistance

**DOI:** 10.3390/ijms21134576

**Published:** 2020-06-27

**Authors:** Anna M. Czarnecka, Ewa Bartnik, Michał Fiedorowicz, Piotr Rutkowski

**Affiliations:** 1 Department of Soft Tissue/Bone, Sarcoma and Melanoma, Maria Sklodowska-Curie National Research Institute of Oncology, 02-781 Warsaw, Poland; piotr.rutkowski@coi.pl; 2 Department of Experimental Pharmacology, Mossakowski Medical Research Centre, Polish Academy of Sciences, 02-106 Warsaw, Poland; 3 Institute of Genetics and Biotechnology, Faculty of Biology, University of Warsaw, 02-106 Warsaw, Poland; ebartnik@igib.uw.edu.pl; 4 Institute of Biochemistry and Biophysics, Polish Academy of Sciences, 02-106 Warsaw, Poland; 5 Small Animal Magnetic Resonance Imaging Laboratory, Mossakowski Medical Research Centre, Polish Academy of Sciences, 02-106 Warsaw, Poland; mfiedorowicz@imdik.pan.pl; 6 Interinstitute Laboratory of New Diagnostic Applications of MRI, Nalecz Institute of Biocybernetics and Biomedical Engineering, Polish Academy of Sciences, 02-109 Warsaw, Poland

**Keywords:** BRAF, MEK, melanoma, MAPK, drug resistance

## Abstract

The common mutation *BRAFV600* in primary melanomas activates the mitogen-activated protein kinase/extracellular-signal-regulated kinase (MAPK/ERK) pathway and the introduction of proto-oncogene B-Raf (BRAF) and mitogen-activated protein kinase kinase (MEK) inhibitors (BRAFi and MEKi) was a breakthrough in the treatment of these cancers. However, 15–20% of tumors harbor primary resistance to this therapy, and moreover, patients develop acquired resistance to treatment. Understanding the molecular phenomena behind resistance to BRAFi/MEKis is indispensable in order to develop novel targeted therapies. Most often, resistance develops due to either the reactivation of the MAPK/ERK pathway or the activation of alternative kinase signaling pathways including phosphatase and tensin homolog (PTEN), neurofibromin 1 (NF-1) or RAS signaling. The hyperactivation of tyrosine kinase receptors, such as the receptor of the platelet-derived growth factor β (PDFRβ), insulin-like growth factor 1 receptor (IGF-1R) and the receptor for hepatocyte growth factor (HGF), lead to the induction of the AKT/3-phosphoinositol kinase (PI3K) pathway. Another pathway resulting in BRAFi/MEKi resistance is the hyperactivation of epidermal growth factor receptor (EGFR) signaling or the deregulation of microphthalmia-associated transcription factor (MITF).

## 1. Introduction

**Figure 1:**
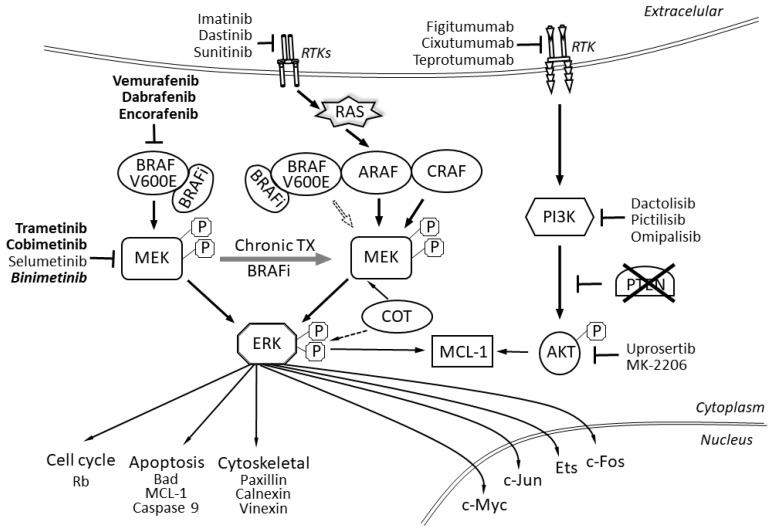
BRAF signaling pathway including abnormal signaling from BRAFV600E mutated proteins. The scheme also shows inhibitors and their targets—drugs approved in melanoma treatment are bolded, we also show other potential inhibitors (drugs in development or registered in other indications). RTK–receptor tyrosine kinase; RTKs–receptor tyrosine kinases; BRAF–proto-oncogene B-Raf; BRAFi–BRAF inhibitors; ARAF–serine/threonine-protein kinase A-Raf; CRAF–RAF proto-oncogene serine/threonine-protein kinase, also known as proto-oncogene c-RAF; PI3K–phosphoinositide 3-kinases; P–phosphoprotein; MEK–mitogen-activated protein kinase kinase; ERK–extracellular-signal-regulated kinase; COT–mitogen-activated protein kinase kinase kinase 8 or serine/threonine-protein kinase cot-1; MCL-1–induced myeloid leukemia cell differentiation protein Mcl-1; AKT–protein kinase B, also known as Akt; Rb–the retinoblastoma protein; Bad–BCL2 associated agonist of cell death; c-Myc–Myc proto-oncogene protein; c-Jun–Jun-related antigen, isoform C; Ets–ETS transcription factor family; c-Fos–proto-oncogene c-Fos.

In terms of molecular pathology, malignant melanomas can be classified into four subgroups on the basis of mutations that are found within the cells: melanoma with mutations in proto-oncogene B-Raf or v-Raf murine sarcoma viral oncogene homolog B (*BRAF*), in the rat sarcoma gene (*RAS*) or neurofibromin 1 (*NF1*) and the so-called triple negative melanomas [1,2,3]. In primary melanomas, mutations most often occur in either the *BRAF* or the *RAS* genes, while concomitant mutations in both were in general not observed except for in nodular melanoma [4]. Mutations in other genes have been reported, with *NRAS* gene (coding N-Ras protein, also called neuroblastoma RAS viral oncogene homolog) mutations being the most common (13–25%) [5,6]. In the most recent data from the Pan-Cancer Analysis of Whole Genomes of 107 sequenced melanomas, 52 *BRAF* mutations, 74 telomerase reverse transcriptase (*TERT*) mutations and 20 tumor protein *p53 gene* (*TP53*) mutations were found and two other genes were frequently mutated: cyclin-dependent kinase inhibitor 2A (*CDKN2A*) (CDK4 inhibitor (P16-INK4 or ARF) (55)) and cyclin-dependent kinase inhibitor 2B (*CDKN2B*) (cyclin-dependent kinases 4 and 6 binding protein or P15_INK4B (27)), but only 10 *NF1* mutations were found [7]. It is also known that some of these mutations (in *BRAF*, *NRAS* and *TERT*) are found both in benign lesions and in melanomas, whereas those in *CDKN2A*, *TP53* and phosphatase and tensin homolog (*PTEN*) are only observed in invasive melanomas [8]. Mutations in the *BRAF* gene are known to be independent of UV light, as mutations induced by UV light are typically C->T nucleotide transitions at the 3′ ends of pyrimidine dimers [9,10]. *BRAF* mutations are not sufficient for melanoma development or progression, as they are already also found in benign nevi. Melanoma oncogenesis is, finally, an effect of many other mutations in different genes, including *TP53*, *PTEN* and *CDKN2A*, and also in the telomerase gene promoter and p16 [1], as malignant melanomas are genetically highly heterogeneous, and they acquire numerous mutations when metastasizing [11]. In fact, the deregulation of the RAS/MAPK (MAPK - mitogen-activated protein kinase) pathway is found in 98% of melanomas [1]. In about 50% of melanoma cases (but only in 7–10% of all neoplasms), a mutation in the *BRAF* gene is present, and 80–90% of these mutations are a missense *V600E* mutation, where the wild type amino acid 600 (a valine) is replaced by a glutamic acid residue. This mutation causes a change in the BRAF protein conformation, increasing its kinase activity, resulting in a constitutive activation of the MAPK/ERK (ERK - extracellular-signal-regulated kinase) pathway (Figure 1). In fact, a substitution of a non-polar valine (V) by a negatively charged glutamic acid (E) at position 600 (V600E) in the *BRAF* kinase [12] blocks it in the activated state in a phosphomimetic manner, which results in constitutive MAPK/ERK downstream signaling and proliferation stimulation and cell survival, promoting melanoma tumor growth [12].

BRAF mutations were discovered in about 50–60% of metastatic melanoma cases [13,14]. Other substitutions in this site are less frequent, including about 7.7% V600K (lysine substitution), 1% V600R (arginine) and leucine and aspartic acid substitutions with a frequency of 0.3% and 0.1%, respectively [15]. Substitutions in other BRAF sites are also found, but altogether represent less than 1%. These mutations are also of important clinical significance, as normal BRAF protein is active as a dimer, but the *V600E* mutation makes it active as a monomer. It is this monomer that various BRAF inhibitors used for melanoma therapy, like vemurafenib, dabrafenib and encorafenib, bind [16,17].

As per the American Cancer Society, the five-year survival rate for stage IV melanoma is 15 to 20%, and historically, the median overall survival (OS) time for patients with advanced, unresectable or metastatic melanoma (stage IV) was only 6–9 months. Currently, this trend has been significantly changed due to the implementation of targeted therapies and immunotherapy. *BRAF*-mutated (*BRAF*+) melanoma is characterized by specific clinical features, including more aggressive biological behavior than *BRAF* wild-type (WT) melanoma. *BRAF*(+) melanoma is known for shorter OS in patients with stage IV disease, shorter than in those with *BRAF* WT disease. Compared with patients with *BRAF* WT melanoma, those with *BRAF*(*+*) are often younger and present with superficial spreading tumors or tumors with nodular histology, developing in anatomical regions without chronic sun damage. The recurrence-free survival (RFS), from the diagnosis of primary melanoma tumor (stage I/II) to the development of distant metastases (stage IV), does not significantly differ between *BRAF*(+) and *BRAF* WT patients, but at the same time, the median OS of patients with newly diagnosed, untreated, metastatic *BRAF*(+) melanoma was historically 5.7 months, and for *BRAF* WT patients, 8.5 months [18]. The clinical implementation of BRAF and MEK (mitogen-activated protein kinase kinase) inhibitor therapies has resulted in dramatic improvements in OS and progression-free survival (PFS) rates in patients with *BRAF*(+) advanced melanoma cases over the last few years [19,20,21,22]. 

Classical systemic methods of melanoma treatment—dacarbazine chemotherapy—was ineffective until targeted therapies were developed. Vemurafenib, the first specific inhibitor acting on the BRAF/MEK pathway, was approved by the Food and Drug Administration in 2011 (Figure 1). Together with vemurafenib, dabrafenib and encorafenib also belong to a group of drugs called BRAF protein inhibitors (BRAFis). Encorafenib differs from other agents in this drug class by a more than 10 times longer dissociation half-life (> 30 h), which results in the extended inhibition of the MAPK signaling pathway and more potent anti-cancer activity [16,23]. Subsequently, the MEK inhibitors (MEKi) cobimetinib, trametinib and binimetinib were registered. In particular, a median PFS of 12.3 months (95% CI, 9.5–13.4 months) for vemurafenib and cobimetinib treatment was reported in the coBRIM trial (ClinicalTrials.gov, number NCT01689519) [15,17]; while a median PFS of 9.3 months for dabrafenib and trametinib therapy in a phase I/II trial, 11 months in the COMBI-d trial and 12.1 in COMBI-v trials were found (COMBI-d ClinicalTrials.gov number, NCT01584648; COMBI-v ClinicalTrials.gov number, NCT01597908) [18,19]. For encorafenib and binimetinib, a median PFS for COMBO450 was recently reported as 14.9 months, while for ENCO300 it was 9.6 months [24]. As per subgroup analyses, PFS on dabrafenib with trametinib treatment is 6.6 months for patients in stage M1c + LDH ≥ 2 × ULN + ECOG (LDH, lactate dehydrogenase; ULN, upper limit of norm; ECOG – Eastern Cooperative Oncology Group; PS - Performance Status)) PS ≥ 1, 7.8 months for M1c + LDH > ULN, 10.6 months for patients with liver metastases [21] and 5.6 months for patients without and 7.2 months for patients with previous local brain therapy [22]. Moreover, the objective response rate (ORR) is 64% for the dabrafenib and trametinib, as per a phase III trial [20], and over 69% for a vemurafenib and cobimetinib combination in a phase III trial. In the coBRIM trial, 15.8% of patients had complete remission (CR), in COMBI-d 18% and in COMBI-v 19% and the median duration of response was 13 months, 12 months and 13.8 months, respectively. The clinical implementation of BRAF and MEK inhibitor therapies (including dabrafenib and trametinib and vemurafenib and cobimetinib) has resulted in dramatic improvements in OS and progression-free survival (PFS) rates in patients with BRAF-mutated advanced melanoma [19]. In particular, three-year OS with BRAFi/MEKis reaches 62% in the most favorable subgroup of patients with normal LDH and < 3 organ sites with metastases, but only 25% in the unfavorable subgroup with LDH > 2x the upper limit of the norm [12]. The four- and five-year OS rates with dabrafenib and trametinib therapy are 30% and 28%, respectively. For four- and five-year PFS, it is 13%. The longest OS is seen in patients with normal LDH (45% at 5 years) and normal LDH with disease in < 3 organ sites (51% at 5 years) [13]. For dabrafenib and trametinib treatment, survival after progression of disease (PD) is the longest in cases with progression in baseline targets or new non-CNS lesions (10.0 months; 95% CI: 7.9–12.0) and shortest in those with new CNS lesions or concurrent progression in baseline and new lesions (median 4.0 months; 95% CI: 3.5–4.9) [23]. Updated data from the COLUMBUS (ClinicalTrials.gov, number NCT01909453, and EudraCT, number 2013-001176-38) trial have shown that median OS is 33.6 months for encorafenib and binimetinib (COMBO450) and 23.5 months for encorafenib 300 mg treatments [24]. 

As the concurrent inhibition of the BRAF and MEK proteins of the MAPK pathway could decrease MAPK-driven acquired resistance, leading to a longer duration of responses, a higher rate of tumor responses and a longer progression-free survival and overall survival, BRAFi/MEKis are used in clinical practice in combinations (vemurafenib and cobimetinib, dabrafenib and trametinib and encorafenib and binimetinib) resulting in median PFS and OS of 12–15 months and 22–33 months, respectively. Unfortunately, about 15–20% of patients with the *BRAF V600E* mutation do not respond to this drug at all [5,17,25]. This may be due, among other reasons, to tumor heterogeneity—the mutation might not be present in all its cells—or to the loss of some tumor suppressor genes, such as *PTEN* and *NF1*, which causes primary resistance to BRAFi/MEKis, as described below [5]. As for mutations in the MEK (MAPK) genes, there are less data, but MEK inhibitors are also used in melanoma therapy, together with BRAF inhibitors to combat fast resistance to BRAFi therapy and decrease the cutaneous side effects related to paradoxical MAPK pathway activation with BRAF inhibitor monotherapy. Primary *MAP2K1* and *MAPK1* mutations are found in only 5.38% and 1.77% of melanoma cases, respectively, and are often inclusion criteria for patients in clinical trials with MEK inhibitors (MEKi) [26]. In clinical practice, the presence of *MEK1*/2 mutations correlates with the presence of liver metastases and their progression [17]. 

In fact, the main cause of resistance to BRAF inhibitors is the reactivation of the MAPK pathway—occurring in 80% of BRAFi-resistant tumors. This may be due to changes in the BRAF protein due to alternative splicing or overexpression. Moreover, as tumor heterogeneity in respect to BRAF V600/wild type BRAF is well documented, the resistance can be due to interactions of BRAFis with wild type BRAF proteins. Alterations in COT (Mitogen-activated protein kinase kinase kinase 8), NRAS, MEK and NF1 can also reactivate this pathway or the activation of alternative pathways takes place [27]. The activation of the MAPK/ERK pathway on BRAFi treatment has been shown to result from the presence of BRAFV600E amplification, the overexpression of the CRAF (RAF proto-oncogene serine/threonine-protein kinase) or COT1 kinases, activating mutations in *NRAS*, *MAP2K1* or *MAP2K*2 or the loss of *NF1*, as well as the expression of splice variants of BRAFV600E [28]. On the other hand, other mechanisms of BRAFi resistance are PI3K/AKT/mTOR-dependent. Mutations in *AKT1*, *AKT3*, *PIK3CA*, *PIK3CG*, *PIK3R2* or *PHLPP1*, as well as *PTEN* loss or the overexpression of multiple receptor tyrosine kinases (RTKs), including epidermal growth factor receptors (EGFRs), insulin-like growth factor 1 receptors, platelet-derived growth factor receptors α and β or fibroblast growth factor receptor 3 have been reported in resistant melanoma tumors [28]. Due to the multiplicity of mechanisms, a very important aim for future research would be to determine the major pathomechanisms of BRAF inhibitors and find biomarkers which would make it possible to foresee the response to treatment before its initiation [29]. 

## 2. The BRAF Protein

BRAF protein function may be reactivated through numerous mechanisms, a frequent one is the amplification of the mutated *BRAF* allele, which leads to the overexpression of the BRAF protein. As a result, the administered dose of the BRAF inhibitor is insufficient to inhibit its activity. This overexpression may also lead to the spontaneous dimerization of the mutated BRAFV600E protein, which causes the reactivation of the ERK signal transduction pathway. This leads to inhibitor resistance, which is described as dose-dependent as it can be overcome in vitro by higher doses of a BRAFi, like vemurafenib [30]. Splicing variants of BRAF are found in approximately 13–30% of resistant melanomas [31,32,33]. Moreover, a splicing variant of BRAFV600E, p61BRAFV600E, has been described in patients with secondary resistance to vemurafenib. This variant forms dimers regardless of activation through the RAS kinase, thus abrogating the effects of BRAF inhibitors, which only act on BRAFV600E monomers. The alternatively spliced BRAF isoforms are due to mutations or epigenetic changes [34,35]. The causes are not always clear. Vido, Le, Hartsough and Aplin [32] have shown that the association of the splice variant with MEK is required for resistance to BRAF inhibitors and that the phosphorylation of serine 729 in the truncated splicing variant of BRAFV600E is increased.

*BRAF* gene amplification has been observed in 20% of BRAFi-resistant tumors [36]. Amplification contributes to the reactivation of ERK [37]. *BRAF^V600E^^/K^* amplification has been reported in about 13% of patients [33].

One of the mechanisms of BRAF inhibitor resistance is due to the microheterogeneity of the tumors—some of the cells are wild type in respect to *BRAF*, while some carry the BRAF V600 mutation. The inhibitors will act on the BRAF V600 monomers, however, in BRAF wild-type cells, the expression of CRAF was shown to be higher and vemurafenib stabilized BRAF–CRAF heterodimers, thus reactivating the MAPK pathway [38]. This is described as the paradoxical activating role for the BRAF inhibitor. 

## 3. The *MAPK*/*ERK* Genes and Signaling

The RAS/RAF/MEK/ERK signal transduction pathway regulates the transcription of genes involved in cell growth, division and differentiation. The signal is transduced by the phosphorylation of successive proteins (Ras–Raf–MEK–ERK), and the final targets are more than 50 transcription factors, including c-Myc and CREB (cyclic AMP response element binding protein), which become activated [38]. This signal transduction pathway is activated by growth factors, hormones and cytokines, which interact with a membrane receptor with tyrosine kinase activity (RTK – receptor tyrosine kinase), leading to its phosphorylation, which in turn transfers the signal to a protein from the RAS (rat sarcoma) family. Activated Ras activates a RAF (Rapidly Accelerated Fibrosarcoma) family protein (ARAF, BRAF and CRAF rapidly accelerated fibrosarcoma proteins), which in turn phosphorylates and activates MEK (mitogen-activated protein kinase kinases MEK1 and MEK2, also known as MAP2K1 and 2 or MAPKK 1 and 2), and MEK phosphorylates and activates the mitogen-activated protein kinase (MAPK/ERK). RAF and ERK are serine–threonine protein kinases and MEK is a serine–tyrosine–threonine kinase. Activated ERK (extracellular signal regulated kinase) migrates to the nucleus where it phosphorylates and thus activates the targeted transcription factors [39]. 

Deregulation at each of the pathway steps may contribute to BRAFi/MEKi resistance, as modifications or mutations downstream of BRAF can occur, including MEK mutations that make this kinase constitutively active and can therefore subsequently activate ERK, but ERK activation may also be induced in a MEK-independent manner (i.e., by COT proteins) [40]. MAPK signaling reactivation, in cases with mutant BRAF amplification or alternative splicing, as well as RAS mutations, are the most common MAPK triggers [41]. In fact, MAPK signaling reactivation was found in up to 70% of melanoma cases upon disease progression [41]. First of all, RAS–RAF–MEK–ERK may become activated by mutations in the genes encoding the MEK1/MEK2 proteins (mitogen-activated protein kinase 1/2). This, downstream of BRAF reactivation of the MEK protein signaling, abrogates the effects of BRAF inhibition as the initiation of the signal at the level of BRAF is no longer necessary for the activation of final target genes [5]. Secondary mutations in both MEK1 and MEK2 are also involved in acquired resistance in melanoma and are found in 7% of BRAFi-resistant melanomas. Known activating MEK1 mutations include Q56P, E203K, C121S and K57E, whereas MEK2 mutations include E207K and Q60P [25,36]. Another gene from this pathway that is often mutated or deregulated in BRAFi-resistant melanomas is *MAP3K8.* The *MAP3K8* gene encodes the MAP3K8 protein (mitogen-activated protein kinase kinase kinase 8, also called COT, EST, ESTF, MEKK8, TPL2, Tpl-2, c-COT and AURA2). The MAP3K8 protein can activate the MAPK/ERK signal transduction pathway through the phosphorylation of MEK. MAP3K8 activates MEK-dependent signaling without RAF signaling and primarily results in ERK activation. An elevated level of COT proteins maintains proliferation in spite of BRAF protein inhibition. MAP3K8-deficient cells are sensitive to BRAFis and present reduced growth and MEK/ERK activity during BRAFi treatment [36,42,43]. Administering BRAF inhibitors in the case of primary *MAP3K8* overexpression further increases the expression of this protein, which in turn stimulates the proliferation of melanoma cells [42,43]. Mutations in *MAP3K8* are present in about 1.5% of all melanoma patients, frequently in Spitz nevi (in about 33% cases) [44]. The usage of MEK and EKR inhibitors has been suggested as a strategy for targeting MAP3K8 melanoma dependence [45]. 

## 4. The *RAS* Gene

The *RAS* gene is one of the most commonly mutated oncogenes in human neoplasms and mutations in *RAS* inhibit GTPase activity and keep the protein in the active GTP-bound conformation. RAS mediates signal transduction downstream from tyrosine kinase membrane receptors to more than 300 Ras-responsive target genes [46]. In BRAFi-resistant melanoma, the upstream reactivation of signal transduction from the cell membrane to MAPK/ERK kinases is caused by the overexpression of tyrosine kinase receptors, which leads to cell division by the activation of ARAF and CRAF kinases instead of BRAF. During treatment with BRAFi/MEKis, cells with the BRAFV600E mutation may acquire resistance to treatment by switching signal transduction to different RAF isoforms (ARAF or CRAF), which causes the reactivation of signal transduction within the ERK pathway [47]. The MAPK/ERK pathway may also be activated due to mutations in the RAS gene. Hyperactivated RAS may phosphorylate the ARAF and CRAF proteins, which compensates for BRAF inhibition and promotes cell division. In melanoma cells, ARAF or CRAF may be overexpressed, while BRAF is blocked. The mutated RAS protein, after binding GTP, does not dissociate to the inactive form bound to GDP and is permanently activated. The mutated protein bound to GTP also promotes BRAFV600E dimerization, the reactivation of the ERK signal transduction pathway and leads to resistance to BRAF inhibitors, as they only bind BRAFV600E monomers [48,49,50].

Moreover, the ERK protein is a negative regulator of the RAS protein; BRAF inhibitors inhibit cell growth by inhibiting the ERK pathway. Blocking signal transduction through the ERK pathway stops RAS regulation, inducing partial RAS activity. Resultant RAS hyperactivation leads to the formation of BRAFV600E dimers. BRAF inhibitors bind to one of the monomers, which leads to the transactivation of the other monomer which is not bound by the drug. Such BRAF activation leads to the partial activation of signal transduction by ERK and contributes to limiting the effectiveness of the treatment [51,52]. 

## 5. The *RAC1* Gene

*RAC1* encodes the Rac Family Small GTPase 1 (RAC1 protein, other names: cell migration-inducing gene 5 protein, Ras-related C3 botulinum toxin substrate 1, Ras-like protein TC25, P21-Rac1). RAC1 is a GTPase that regulates the cell cycle, cellular adhesion, cell mobility (by acting on the cytoskeleton) and cell differentiation [53]. The P29S mutation in the *RAC1* gene is found in 3.3% of melanomas [54], but in as many as about 20% of patients not responding to treatment with BRAF inhibitors [55]. The presence of this mutation in melanoma cell lines causes resistance to BRAF inhibitors [54]. The *RAC1^P29S^* mutation was shown to activate the SRF/MRTF (SRF - serum response factor, MRTF - myocardin-releated transcription factor) pathway, as well as PAK (serine/threonine-protein kinase PAK 1) and AKT. In consequence, a melanocytic to mesenchymal phenotype transition was observed [56]. The presence of this mutation correlates positively with the mitotic index, the size of the tumor and also the occurrence of metastases [54].

## 6. The *PTEN* Signal Transduction Pathway

The *PTEN* (phosphatase and tensin homolog) gene is a suppressor gene—the protein it encodes, phosphatidylinositol-3,4,5-trisphosphate 3-phosphatase (PTEN, MMAC1), is involved in cell cycle regulation. PTEN catalyzes PIP3 dephosphorylation in the 3′ position of the inositol ring, which inhibits the PI3K/AKT signal transduction pathway, and as a result, blocks cellular proliferation [57]. 

Loss of a functional *PTEN* gene is observed in 10–35% percent of melanoma cases and is one of the most common causes of resistance to BRAF inhibitors [36,58]. The loss of PTEN protein expression leads to the constitutive activation of the PI3K/AKT signal transduction pathway, leading to cell proliferation, growth and the inhibition of apoptosis. The inhibition of apoptosis in the case of *PTEN* loss is induced through the Bcl-2-like protein 11 (BIM, encoded by *BCL2L11* gene) [58]. 

Patients with deletions and/or mutations in *PTEN* achieve shorter PFS on BRAFi therapy (18 weeks) in comparison to patients without such mutations, but this difference was not statistically significant (32.1 *weeks*; *p* = 0.066). Moreover, a trend towards an association between low expression of *PTEN* and low ORR in patients treated with BRAFis was reported [59,60]. A combination of BRAFis and PI3K inhibitors has been suggested as a therapy to overcome PTEN signaling abnormalities [41].

## 7. The NF1 Signal Transduction Pathway

The *NF1* gene encodes neurofibromin (also called neurofibromatosis-related protein NF-1), which is a member of the GTPase-activating group of proteins. Neurofibromin regulates cell proliferation, differentiation and survival. It is a negative regulator of RAS, the first protein of the MAPK signal transduction pathway and neurofibromin inactivates RAS–GTP through catalyzing hydrolysis of RAS–GTP to RAS–GDP. In melanocytes, neurofibromin also regulates melanogenesis—*NF1* loss results in the enhanced production of melanin [61]. The absence of functional neurofibromin results in the enhancement of several signaling pathways, including MAPK and PI3K, and subsequently promotes cell proliferation and cell survival [62].

The *NF1* gene was found, to be after *BRAS* and *NRAS*, to be the third most frequently mutated in melanoma [63]. Various aberrations within the *NF1* gene were found in 17% of samples in the Melanoma Genome Project report [3]. These aberrations included mostly point mutations (most frequently nonsense mutations or missense mutations). *NF1* mutations were reported to co-occur with mutations in the *RASA2* gene [3,63] that is another tumor suppressor gene inactivated in ca. 30% of melanomas and associated with poor prognosis [64]. Other mutations frequently associated with NF1 loss in melanoma occur in *PTPN11*, *SOS1*, *RAF1*, *SPRED1* and other genes. Some data suggest that these mutations may act synergistically in melanoma [65,66].

The loss of a functional product of the *NF1* gene contributes to one of the mechanisms of BRAFi resistance in melanoma. The resulting constitutive activation of the MAPK signaling pathway is not suppressed by BRAF inhibitors [67]. Suppression or the loss of neurofibromin seems to be a frequent event in tumors exposed to BRAF/MEK inhibitors [68]. It seems that mutations in the *NF1* gene co-occur with *BRAF* mutations in melanomas and may also play a role in acquiring resistance to BRAF inhibitors [68].

## 8. The EGFR Signal Transduction Pathway

Epidermal growth factor receptor (EGFRErbB-1; HER1), a transmembrane protein that is a receptor tyrosine kinase for members of the epidermal growth factor family (EGF family) ligands, is reportedly involved in the autocrine growth of melanoma cells [69]. In BRAFi-resistant cell lines and in resistant tumor biopsies, the upregulation of EGFR expression was reported. The overexpression of EGFR is known to derive from the demethylation of *EGFR* regulatory DNA elements. EGFR signaling activates the PI3K/AKT pathway. As a result, resistant cells show high spontaneous migration and invasion with the highly increased activity of MMPs: MMP2, MMP9 and MMP14 [70]. The hyperactivation of the EGFR–SRC family kinase signal transduction and the subsequent activation of the signal transducer and activator of transcription 3 (STAT3) pathway signaling was also reported [28]. It was also confirmed that EGFR overexpression induces BRAFi resistance without ERK induction and represents a MAPK-independent resistant pathway [71]. After EGRF activation, the complex formed by the Grb2 and Sos proteins binds directly or through the adaptor protein Shc with specific tyrosine residues on the receptor. This causes conformational changes in the Sos protein, which can recruit and activate Ras–GDP. Subsequently MAPK kinases activated by ERK migrate to the nucleus and phosphorylate specific transcription factors, such as Elk1 and C-myc, inducing cellular proliferation [72]. A decrease in the activity of sex determining region Y-box 10 (SOX10) described in some melanomas may lead to signalization through TGF-β, which in turn leads to an increase in the expression of EGFR and the receptor of the platelet-derived growth factor (PDGFRB) [5,73]. Nevertheless, in BRAFi-resistant EGFR overexpressing melanoma cells, EGFR is functional but usually inactive. Therefore, basal EGFR expression may not be used as a predictive biomarker for BRAFi/MEKi treatment [74]. At the same time, EGFR expression was significantly correlated with metastatic disease status and therefore suggested as prognostic factor in melanoma [69].

## 9. The HGF Signal Transduction Pathway

The hepatocyte growth factor (HGF) is a factor for cell growth and mobility and a morphogenic factor. Pleiotropic HGF activity is mediated by its receptor, a transmembrane tyrosine kinase encoded by the cMet protooncogene - tyrosine-protein kinase Met or hepatocyte growth factor receptor (HGFR) [75]. HGF signaling is responsible for BRAFi resistance development in at least two mechanisms. Primary resistance to BRAF is induced due to HGF secretion by stroma cells (i.e., fibroblasts) within the tumor and its paracrine signaling to melanoma cells. HGF can bind RTK on the surface of melanoma cells, which increases intracellular signaling, which promotes RAS expression, finally leading to the activation of the MAPK signal transduction pathway. In fact, HGF secretion leads to the activation of the HGF receptor—the MET protein—and the subsequent downstream activation of the MAPK/ERK and PI3K/AKT signal transduction pathways. The activation of these pathways also leads to the maintenance of proliferation in the presence of BRAF inhibitors [76,77]. Moreover, HGF is known to contribute to the development of resistance to BRAFi treatment by decreasing the expression of genes encoding pro-apoptotic proteins [76,78,79]. 

## 10. The PI3K/AKT Signal Transduction Pathway

The PI3K/AKT/mTOR pathway is an intracellular signaling pathway important in the regulation of cell proliferation, quiescence and survival during cellular stress, and when activated, provides a growth advantage, metastatic potential and angiogenesis induction in melanoma tumors [76]. Mutations leading to an increase in PI3K/AKT pathway activity have been identified in 22% of melanomas with acquired resistance to BRAF inhibitors. An increase in AKT protein expression has been demonstrated several days after administering a BRAF inhibitor [41]. 

The PI3K/AKT signal transduction pathway communicates with the ERK pathway, therefore the inhibition of one of these two pathways can increase the activity of the other one. The blockage of ERK signaling leads to the adaptative overactivity of PI3K/AKT, which compensates for BRAF inactivation and results in acquired BRAFi resistance. Abnormal PI3K/AKT signaling is a common phenomenon in melanoma cells and it actually causes resistance through the stimulation of alternative downstream pathways in the melanoma cell, which decreases dependence on ERK signaling for proliferation [41,77,80]. The initial hypothesis resulting from preclinical data was that, during treatment with BRAFi/MEKis, there is strong selection pressure in respect to cells with gain of function mutations, leading to increased PI3K/AKT pathway activity in the presence of MAPK pathway activation. The assumption was made that melanoma cells with such mutations would continue to divide, as they have an advantage in respect to survival and proliferation when their metabolism is not affected by BRAF inhibition. This increased proliferation of cells with an activated AKT pathway may explain the presence of a large tumor mass and rapid progression in patients who responded to BRAF inhibition and then developed secondary resistance. Moreover, the PI3K/AKT pathway is activated by growth factors which bind RTK, such as PDGFR-β and IGF-1R. The high expression of the RTK receptors on the surface of melanoma cells is linked with acquired vemurafenib resistance both in vitro and in vivo. PDGFR-β and IGF-1R expression, which lead to PI3K/AKT signaling, prevent apoptosis and favor cell survival. Moreover, mutations which activate PI3K and AKT can increase signaling in the AKT pathway, and again increase antiapoptotic signals and upregulate key genes involved in proliferation. In particular, activated AKT phosphorylates 9000 substrate proteins, including murine double minute-2 (MDM-2), p21 cyclin dependent kinase inhibitory protein, Cip1 (p21), X-linked inhibitor of apoptosis (XIAP), apoptotic signal kinase 1 (ASK1), B cell leukemia/lymphoma-2 interacting mediator of cell death (Bim), B cell leukemia/lymphoma-2 associated death agonist (Bad), forkhead box O3 (Foxo3a) and many others [81]. These changes allow the survival and replication of melanoma cells regardless of BRAF inhibition, which clinically causes acquired resistance [5,80,82].

## 11. Cyclin and Cyclin-Dependent Kinase Genes

The *CCND1* (*BCL1*) gene encodes cyclin D1 (also called B-cell lymphoma 1 protein), a key protein for cell cycle regulation (G1/S phase transition), and is also responsible for the BRAFi resistance of melanoma cells [83]. Cyclin D1 overexpression is sufficient to increase melanoma BRAFi resistance but this phenomenon is even more enhanced when cyclin D1 and CDK4 are concurrently overexpressed [83]. In fact, cyclin D1 regulates proliferation while binding to CDK4 and CDK6, which in turn activates retinoblastoma protein (pRb) and promotes cell cycle progression [84]. *CCND1* amplification has been observed in 20–38% of melanoma samples, which indicates that a large group of patients is potentially resistant to BRAFi and could benefit from treatment with a CDK4/6 inhibitor [84,85,86]. In cells in which the *CCND1* gene has been amplified, there is an increase in cyclin D1 production, and as a consequence, BRAF inhibition is not sufficient to inhibit proliferation [83]. In a mouse model, it was shown that CDK4-6 inhibitors alone—regulating the G1-S transition—failed to induce objective responses, but when combined with BRAFi/MEKis, complete responses may be achieved in 30% [84].

## 12. The *MITF* Gene

The *MITF* gene encodes melanocyte-inducing transcription factor (other names are microphthalmia-associated transcription factor or melanogenesis-associated transcription factor), a “master” regulator of the development and function of melanocytes. MITF controls a wide range of biological processes, including DNA repair, senescence and cell metabolism, as well as cell survival, differentiation and proliferation [87]. One of its actions is a pro-survival effect. MITF exerts its functions through regulating the expression (MITF binds to DNA as a homodimer or heterodimer with TFEB or TFE3) of several genes, including *TYRP1*, *GPNMB*, *TYR*, *BCL2* and *CDK2*. The regulatory actions of MITF are fine-tuned by several types of post-translational modifications, including ubiquitination, sumoylation and phosphorylation, which affect the function of MITF. For example, MITF sumoylation was shown to affect cell senescence and seems to be associated with development of melanoma [88].

Melanoma cells with *BRAFV600E* mutations are characterized by altered *MITF* expression and activity [89]. Amplifications of the *MITF* gene were demonstrated in 10% of samples in a study by the Melanoma Genome Project group and the amplifications were noted in all the melanoma subtypes included in this study [3]. However, different levels of expression of MITF are probably linked to different behaviors of malignant cells. High levels will promote differentiation, while moderate expression will drive proliferation and low expression will drive invasion [90].

This complicated multifaceted role of MITF in melanoma is also reflected in its role in the mechanisms of resistance to BRAF inhibition. Both the expression and loss of MITF may contribute to BRAFi resistance [89,90]. The overexpression of MITF was shown to reduce the therapeutic effect of BRAF inhibitors [91,92] or MEK inhibitors [41], due to the pro-survival functions of MITF. One of the proposed mechanisms is the stimulation of cAMP pathway signaling [91]. 

On the other hand, MITF loss was shown to predict early resistance to targeted therapies, including BRAF inhibitors, and seems to be a common event in acquired resistance to BRAF inhibitors in melanoma [93]. It was observed that MITF downregulation was correlated with the upregulation of AXL (tyrosine-protein kinase receptor UFO). Low MITF/high AXL expression would enhance drug resistance. Even though such an expression pattern can be observed in cells unexposed to BRAF inhibitors, a low MITF/high AXL pattern would be increased as the melanoma progresses [93,94].

## 13. Epigenetic Mechanisms of Resistance to BRAF Inhibitors

Epigenetic or non-genetic mechanisms of regulating gene expression include DNA methylation, the processing of mRNA, RNA stability, microRNAs (miRNAs), nucleosomal positioning and the remodeling of chromatin. Both genetic and epigenetic mechanisms may contribute to acquiring drug resistance. Moreover, these two types of mechanisms may interplay to promote a progenitor-like phenotype and tumorigenesis, as well as tumor heterogeneity [95]. The main epigenetic mechanisms that we cover in this subsection are associated with DNA methylation and histone modifications.

DNA methylation (the presence/transfer of methyl groups covalently bound to cytosine bases) is catalyzed by DNA methyltransferases (DNMTs). In particular, the so-called CpG sites, i.e., dinucleotide regions with a cytosine base preceding a guanine base with a phosphodiester bond shared between these two dinucleotides, are present in the coding regions and are usually methylated. So-called CpG islands, regions rich in CG dinucleotides that are present mostly at gene promoter regions, normally remain unmethylated. In malignant cells, the pattern of methylation is changed globally, DNA methylation is usually decreased (hypomethylation) with some site-specific hypermethylation [95]. The role of methylation in melanoma is not clear. On the one hand, the tumors were shown to contain several thousand hypermethylated regions and according to a 2015 DNA methylation landscape study, 179 tumor-specific methylation sites were identified that could be melanoma biomarkers [96]. On the other hand, it seems that there is no correlation between the number of methylated sites and *BRAF* mutations [96,97]. The higher expression of DNMT3B was shown to correlate with melanoma progression [98] and a recent study also demonstrated a correlation between higher methylation and worse prognosis [97].

A study by El Amran et al. [99] suggests that histone modifications, rather than changes in DNA methylation in melanoma, contribute to drug resistance. Histone modifications are driven by histone-modifying enzymes of three major categories: (1) writers that deposit the post-translational modifications; (2) readers that recognize histone post-translational modifications and facilitate the recruitment of protein complexes; and (3) erasers, that is, enzymes that remove post-translational modifications (e.g., histone demethylases and histone deacetylases, HDACs) [100]. The exposure of melanoma cells to BRAF and MEK inhibitors induced the upregulation of histone methyltransferases (SETDB1 and SETDB2) [99], as well as the overexpression of histone demethylases (KDM6A, KDM6B, KDM1B, JARID1A, JARID1B) [101,102,103]. Melanoma cells resistant to MAPK inhibitors were also shown to be characterized by the reduced expression of SIRT6, a histone deacetylase, and in consequence, the activation of the AKT pathway [104].

## 14. Treatment Regimens Enabling the Overcoming of BRAFi/MEKi Resistance

Although the inhibitors of the MAPK pathway are associated with high objective response rates, most responses are not durable [105]. There are several clinical approaches to prolong the activity of BRAF/MEK inhibitors and to overcome the resistance mechanisms either as combination treatment (Table 1) or sequential treatment (Table 2). The first example, which has started in clinical trials, are combinations with other targets as inhibitors of metabolism, immune surface receptors, epigenetic modulators and agents blocking other oncogenic kinases (e.g., triplet combination of BRAF plus MEK inhibitor plus CDK4/6 inhibitor or inhibitors of the PI3K/AKT/mTOR pathway). The CDK4/6 inhibitors palbociclib (PD-0332991), voruciclib (P1446A-05) and ribociclib (LEE011) are currently being evaluated in combination with BRAF and MEK inhibitors in phase I/II studies [106]. Buparlisib, a pan-PI3K inhibitor, is being evaluated in combination with the BRAF inhibitor vemurafenib (NCT01512251) and MEK inhibitors trametinib (NCT01155453) and binimetinib (NCT01363232). The most advanced clinical trials concern combined or sequential immunotherapy and targeted therapy. Immune checkpoint inhibitors (ICI) are associated with more durable responses, but response rates are lower. The combination of BRAF/MEK inhibition with ICI demonstrates complementary activity in the clinic, so the combination of the two approaches is appealing. It is further supported by preclinical and translational data proving the immune-mediated anti-tumor effects and microenvironment changes of BRAF pathway inhibitors [107,108,109,110]. Early phase studies have demonstrated promising activity in melanoma patients with the manageable safety profile of such combinations [111,112,113,114,115,116]. The advanced phase studies focusing on triple combinations of anti-PD-1/anti-PD-L1 or sequential therapy are ongoing. The summary of these trials is shown in Table 1 and Table 2. In 2020, the early positive results of the IMspire150 trial with 514 untreated advanced melanoma patients [117], who were randomly assigned 1:1 to one of two treatment arms, treatment with vemurafenib, cobimetinib, and atezolizumab or treatment with vemurafenib, cobimetinib and placebo, were disclosed. The data showed a statistically significant and clinically meaningful improvement in investigator-assessed PFS (median PFS for patients in the atezolizumab arm was 15.1 months versus 10.6 months in the placebo arm). There was also significant improvement—the duration of response was 21.0 months for the atezolizumab arm compared with 12.6 months for the placebo arm.

## 15. Conclusions

Melanoma cells become resistant to BRAFi after several months of therapy. This can be due to mutations which increase the frequency of RAF dimerization and promote MAP/ERK signaling. In fact, the most common pathomechanism of BRAFi/MEKi resistance is the reactivation of the BRAF/MEK pathway or another pro-proliferation signal transduction pathway. First of all, *BRAF* gene expression deregulation may result in BRAFi resistance development. In fact, the overexpression of mutated BRAFV600E proteins, including gene amplification, results in BRAFi inefficiency. An increased number of copies of the BRAFV600E protein in the cell (due to an increase in the number of copies of the gene) favors BRAFV600E dimerization and results in the reactivation of the ERK pathway [33,37]. Moreover, the splicing variant of BRAFV600E, p61BRAFV600E, caused by mutations or epigenetic modifications, can form dimers independently of RAS, making BRAF inhibitors ineffective, as they only block monomeric BRAFV600E [31,32]. The overexpression of the *BRAF* gene leads to the formation of large amounts of the BRAF protein, and this may result in dimerization. Surprisingly, to the best of our knowledge, no secondary mutations in BRAF have been found in melanomas, though there is an example of a BRAF secondary mutation in a V600E brain tumor [118], namely L514V, and in this case it conferred resistance to dabrafenib. Moreover, BRAF inhibitors bind one BRAF and transactivate the other one, decreasing the effectiveness of treatment with BRAF inhibitors. 

Dimerization may also be affected indirectly by mutations in the *RAS* gene [5]. Mutations activating the *RAS* gene are pro-proliferative, as mutated RAS–GTP becomes constitutively active, increases BRAFV600E dimerization, reactivates the ERK pathway and also promotes resistance to BRAF inhibitors, which only block monomeric BRAFV600E [29].

The activation of upstream proteins, like other RAFs, e.g., ARAF and CRAF, induces BRAFi resistance. With the activation of alternative isoforms of the RAF protein, BRAFV600E, melanoma treated with BRAF inhibitors can become resistant through flexible switching between RAF isoforms capable of ERK pathway signaling, increasing ARAF or CRAF expression. Moreover, the BRAF inhibitor inhibits tumor growth by inhibiting the ERK pathway, and this in turn inhibits the negative feedback inhibition of ERK on RAS, which partially restores RAS activity, leading to the formation of BRAFV600E dimers induced by RAF [29]. 

Activating mutations in *MEK1*/*MEK2* make the blocking of BRAF ineffective, as MEK reactivation means that the MAPK/ERK pathway can still transduce the signal below BRAF, regardless of its inhibition [25,36]. The overexpression of the COT protein, probably due to gene amplification or hitherto unidentified mechanisms, can reactivate MEK in the presence of BRAF inhibition, stimulating ERK signaling and the development of resistance to BRAFis [42,43]. Finally, the reactivation of MAP/ERK pathway-dependent transcription factors may result from downstream ERK activation and the loss of the inhibitory function of the ERK kinase [29]. 

The PI3K-AKT-mTOR pathway may also become activated and promote melanoma cell proliferation. The activation of the PI3K/AKT signaling pathway thus incorrect PI3K/AKT signaling is a common characteristic of melanomas. Blocking ERK signaling can lead to adaptive PI3K/AKT activity, which compensates for BRAF inhibition and promotes resistance. Additional mechanisms of resistance to BRAFis include the upregulation of the PI3K/AKT/mTOR signaling axis, resulting from mutations in *AKT1*, *AKT3*, *PIK3CA*, *PIK3CG*, *PIK3R2* or *PHLPP1* genes [28]. Mutations in the *PI3K*/*AKT* genes increase AKT signaling, which provides antiapoptotic signaling and increases the expression of key proliferation genes, providing the cell with survival signals independent of BRAF. The expression of α-smooth muscle actin (SMA), N-cadherin, vimentin and fibronectin is also upregulated in BRAFi-resistant cells [70].

With BRAF blocked, tumor cells can overexpress RTK, leading to permanent PI3K/AKT signaling. In inhibitor-resistant melanoma cells with the BRAF mutation, platelet derived growth factor receptor (PDGFRbeta) overexpression is observed, but other changes are also present. They include the increased expression of the insulin-like growth factor 1 receptor (IGFR1), which causes PI3K—AKT—mTOR pathway reactivation. In some resistant cells, the overexpression of the epidermal growth factor receptor (EGFR) or platelet-derived growth factor receptor α and β, is observed [23]. The phenomena which are responsible for resistance have not been determined in 41.7% of patient samples [119]. The PI3K/AKT pathway is activated by growth factors which bind receptor tyrosine kinases (RTKs). RTK signaling can also bypass mutant BRAF and activate ERK via RAS [28]. At the same time, activating mutations in the *PI3K*/*AKT* genes increase AKT signaling, which increases antiapoptotic signaling and increases the expression of key proliferation genes, providing the cell with survival signals independent of BRAF. An increase in RTK or its ligand expression, autocrine tumor cell production and paracrine signaling from tumor stroma promote BRAFi resistance [70].

## Figures and Tables

**Table 1:** The summary of the most important current clinical trials with targeted therapy and immune checkpoint inhibitors in combination treatment.

Study/Phase	Key Inclusion Criteria and Drug Combinations	Primary Endpoint	Key Secondary Endpoints
**TRILOGY** **Phase III**	Stage IV (metastatic) or unresectable stage IIIc (locally advanced) melanoma *BRAF*V600 mutation positive ECOG PS of 0–1 Vemurafenib + cobimetinib +/− atezolizumab	• PFS	Percentage of patients with OR DOR OS
**COMBI-i** **Phase III**	Unresectable or metastatic melanoma *BRAF*V600 mutation positive ECOG PS ≤ 1 Dabrafenib + trametinib +/− spartalizumab	• PFS	OS ORR DOR DCR
**KEYNOTE-022** **Phase I/II**	Unresectable stage III (advanced) or stage IV (metastatic) melanoma *BRAF*V600 mutation positive ECOG PS 0–1 Dabrafenib + trametinib +/− pembrolizumab	• PFS	• ORR
**IMMU-TARGET** **PHASE I/II**	Locally advanced, unresectable/metastatic, treatment naive melanoma Stage IIIB, IIIC or IV with no active brain metastasis *BRAF*V600 mutation positive ECOG PS 0–1 Encorafenib + binimetinib +/− pembrolizumab	PFS at 12, 18 and 24 months	At 24 months ORR OS
**TRIDENT**	Metastatic melanoma (stage IV) or unresectable stage III melanoma that have progressed to prior PD-1 directed therapy; patients with BRAF or BRAF wild type are eligible Nivolumab + trametinib +/− dabrafenib	• ORR	OS PFS CR, PR, SD

**Table 2:** The summary of the most important current clinical trials with targeted therapy and immune checkpoint inhibitors in sequential treatment.

Study/Phase	Key Inclusion Criteria and Drug Sequence	Primary Endpoint	Key Secondary Endpoints
**SWITCH** **Phase III**	Stage III (unresectable) or stage IV disease Presence of *BRAF*V600E or V600K mutation in tumor tissue prior to enrolmen ECOG PS 0–1 Nivolumab + ipilimumab with nivolumab maintenance followed by dabrafenib + trametinib at PD or dabrafenib + trametinib followed by nivolumab + ipilimumab with nivolumab maintenance at PD	• OS	PFS Response rate Safety
**SECOMBIT** **Phase II**	Stage III (unresectable) or stage IV melanoma Treatment-naïve patients Presence of *BRAF*V600E or V600K mutation in tumor tissue prior to enrolment ECOG PS 0–1 Encorafenib + binimetinib followed by nivolumab + ipilimumab with nivolumab maintenance at PD or nivolumab + ipilimumab with nivolumab maintenance followed by encorafenib + binimetinib at PD or encorafenib + binimetinib for 8 weeks with a switch to nivolumab + ipilimumab with nivolumab maintenance followed by encorafenib + binimetinib at PD	• OS	PFS 2/3-YS Best ORR DOR Safety QoL
**EORTC EBIN** **Phase II**	Stage III (unresectable) or stage IV melanoma Treatment-naïve patients Presence of *BRAF*V600E or V600K mutation in tumor tissue prior to enrolment ECOG PS 0–1 Nivolumab + ipilimumab with nivolumab maintenance or encorafenib + binimetinib for 12 weeks with a switch to nivolumab + ipilimumab with nivolumab maintenance followed by encorafenib + binimetinib at PD	• PFS	OS CR ORR PFS2Safety
